# The medicalisation of menstruation: a double-edged sword

**DOI:** 10.12688/wellcomeopenres.24017.2

**Published:** 2025-09-26

**Authors:** Andrea Ford, Jessica Campbell, Katie F.M. Marwick

**Affiliations:** 1 The University of Edinburgh Usher Institute of Population Health Sciences and Informatics, Edinburgh, Scotland, UK; 2 The University of Edinburgh Centre for Clinical Brain Sciences, Edinburgh, Scotland, UK

**Keywords:** menstrual cycle, medicalisation, gender, history of psychiatry, anthropology, hormone replacement therapy, period products

## Abstract

**Background:**

The intersection of menstrual and mental health is an under-researched area which is gaining attention from the public, researchers and clinicians. However, there may be unintended risks associated with this increased attention. Here we review some historical and cultural aspects of the well-intentioned medicalisation of menstruation and discuss their impact on gender (in)equality.

**Aims:**

To critically integrate lessons from the past into current momentum around improving women’s healthcare.

**Method:**

Narrative review with interdisciplinary authorship including psychiatry, medical anthropology, and history.

**Results:**

We explore three exemplars. 19 ^th^ century psychiatry identified menstruation as a time of risk for mental disorder, which recognised relevant aspects of female experience but used this to argue against female education. The 20 ^th^ century discovery that hormone replacement therapy can ease menopausal symptoms has helped many, but aggressive marketing may increase gender-based stigma regarding female identity, the experience of menopause and aging. Lastly, the introduction of menstrual leave and menstrual product policies aims to ease the burden on those who menstruate, but may have the paradoxical effect of portraying those who menstruate as a burden themselves.

**Conclusions:**

Menstruation research occurs in the context of historical and ongoing sexism and the process of menstruation is currently highly stigmatised in many cultures. This makes the process of change complex and rife with ‘double edged swords’ whereby well-intentioned initiatives can have unintended effects. Researchers and clinicians in this area should be aware of how attention to menstruation and gendered difference can be misconstrued and used to inadvertently further gender-based disparities.

## Plain Language Summary

Sometimes well-intentioned ideas can have unexpected negative consequences. In this review, we discuss three examples of such ‘double edged swords’ when medicine and policy pay attention to women’s bodies. First, we explore how 19th century psychiatry identified the onset of menstruation as a time of risk for mental disorder, leading to suggestions that women should not be educated or engage in mental activity. Second, we revisit the advent of hormone replacement therapy (HRT) in 20th century menopause care, which has improved the wellbeing of many. Yet, HRT has been aggressively marketed to correct the perceived unstable behaviour of menopausal women, tying into broader prejudices about aging. Third, we consider 21st century policies for menstrual leave and the provision of free period products, which take steps towards reducing great stress for many menstruating people. Yet, they do so by singling out menstruators as in need of special help, instead of addressing poverty and health-related leave in more general terms that recognise how people of all sorts have different energy levels, discomforts, and hygiene needs. We conclude with suggestions for moving forward thoughtfully with these double-edged swords in mind.

## Introduction

It is heartening to see the rise in public attention to menstruation, menopause, and mental health. This has been accompanied by the enthusiastic decrying of stigma and taboo, and outrage against medical misogyny, research inequities, and clinical dismissal. For example, Cosmopolitan dubbed 2015 the ‘year the period went public’ ^1^ and some in the medical community are characterising menstruation as a ‘fifth vital sign’ ^2^. Celebrities have ‘gone public’ about their experiences of menstrual health, and a flood of popular books in US and UK markets address menstrual wellness and specifically target people dissatisfied with medical treatment of menstrual disorders. We celebrate this, and yet recognize that it is not as straightforward as it might seem. Yes, more research should be done. Yes, accounting for sex-differences is long-overdue and will benefit men as well as women. Yes, more options should be offered to menstruating people who present with complaints. And alongside this, the social consequences bear thinking about, because anytime attention is paid to women’s bodies, there is the risk of slipping into regressive, controlling social paradigms. It is a classic problem in feminist theory: does drawing attention to women’s difference help or hinder efforts to improve their social position? As researchers and clinicians, we urge caution alongside this welcome enthusiasm.

Sometimes well-intentioned ideas can have unexpected negative consequences. In this review, we discuss three examples informed by history, followed by reflections on the future. First, we explore how 19 ^th^ century psychiatry identified the onset of menstruation as a time of risk for mental disorder, but then interpreted this as indicative of frailty. This led to exhortations that women should not be educated or engage in mental activity as it would compromise their reproductive energies. Second, we revisit how the 20 ^th^ century found the menopause being pathologized with the advent of hormone replacement therapy (HRT). HRT has improved the wellbeing of many, yet has been aggressively marketed to correct perceived irrationality and emotional instability of the post-reproductive woman, tying into broader prejudices about aging. Third, we consider 21 ^st^ century policies for menstrual leave and provision of free period products, which take steps towards alleviating considerable stress for many menstruating people. Yet, they do so by singling out menstruators as in need of special accommodations, instead of addressing poverty and health-related leave in more general terms that recognize how people of all sorts have fluctuating energy levels, discomforts, and hygiene needs. We conclude with suggestions for moving forward thoughtfully with these double-edged swords in mind.

Before launching into the examples, we want to briefly touch on some social scientific and philosophical concepts on which our analysis draws: medicalisation, categorisation, and equity. Medicalisation refers to addressing problems which have social and cultural components as though they were primarily medical problems (see, e.g., 3), sometimes pathologising them (treating differences as though they are diseases). Medicalisation is a continuous process that occurs on multiple levels, and demedicalisation can occur as well ^4^, for example removing homosexuality from the DSM (Diagnostic and Statistical Manual of Mental Disorders). One of the earliest attempts to define medicalisation argued that the process requires both the medical *definition* of a social problem and medical *jurisdiction* over that problem: ‘defining behavior as a medical problem or illness and mandating or licensing the medical profession to provide some sort of treatment for it’ ^5^. In 1980, Crawford identified how the pursuit of health in everyday life not only is medicalised but individualised, putting the onus of problems and responsibility for solutions on individuals when they would more appropriately be considered issues of general social wellbeing, for example, nutrition ^6^.

Categorisation is key to how the double-edged swords we discuss work, particularly labels and categories given by scientists and medical professionals (e.g. diagnostic categories) ^1^. Recognising and naming problems that are particular to a *kind* of person inevitably creates and reinforces that kind of person – for example, characteristics associated with “a perimenopausal woman” become cultural knowledge that in turn is referenced by people who menstruate and are nearing a certain age bracket to understand and interpret their experience. Hacking explains how this “looping effect” in fact changes the reality that the category sought to name and understand in the first place ^7^. Another aspect of this phenomenon is that people treat other people *as if* they are that kind of person and have the features associated with that category, again influencing and creating the social reality such categories are meant to describe ^8^. In a stereotyped example, a woman is upset about something, her boyfriend (whether considerately or impatiently) wonders if she is having PMS, the woman (whether resentfully or appreciatively) comes to understand her anger as a possible “symptom” of PMS, perhaps seeking psychological or psychiatric treatment for it and being wary of her emotions “at that time of the month”, and the category of “a PMS-ing woman” as someone angry and irritable is reinforced. The practical importance of this phenomenon is demonstrated by findings that negative constructions of PMS by partners are associated with greater PMS-related distress for the woman ^9^. Further, a randomised controlled trial that found that treating PMS with CBT delivered to couples was more effective in some domains than CBT delivered to the affected individual only ^10^, suggesting that relationship dynamics may at least in part contribute to PMS symptoms ^11^.

Once in existence, these social categories can be used to various ends. They can be weaponised, used to dismiss and belittle people or subject them to harmful or degrading processes. This process builds on substantial cultural and historical sexism: 91% of men and 86% of women were found to be sexist when surveyed in 75 countries as part of the nationally representative World Values Survey between 2005 and 2014 ^12^. Sexism was defined by holding at least one bias against women, for example, 36% believed men have more right to a job than women and 50% thought men make better political leaders than women. Discomfort with women in political and corporate leadership roles has increased since 2021 in G7 countries ^13^. This is the backdrop against which research into women’s health is performed. Discriminatory interpretations are the norm. While our focus here is on gender, it is important to mention that the weaponisation of categories takes place intersectionally, and that people associated with more than one ‘kind’ of person can experience different and worse treatment, a problem that is particularly salient at the intersection of gender and race (see, e.g., 14, 15). An historical and cultural perspective on categories can help us take distance from those we take for granted, and be wary about the ends to which such categories might be used. This is one of the things we hope to demonstrate in this piece.

Lastly, equity adds nuance to what it means to be ‘equal’. While equality implies that people are treated the same, equity implies that they experience the same outcome. A classic example is that while one toilet each for men and women is equal, three toilets for women and one for men is equitable as the queue for the women’s toilets is three times as long. An updated version might propose individual non-gendered toilets as a solution that is both equal and equitable (with enclosed sinks allowing for hygiene needs of various sorts to be addressed privately). However, gender-neutral toilets are sometimes viewed as less clean, related to different genders’ socialization around tidiness ^16^. The difference between equality, equity, and justice has been discussed in the context of gynaecological surgeons and gender ^17^, while broader feminist literature both questions and upholds the goal of gender equality (e.g. 18, 19). Attempts by women themselves to downplay biological differences from men, for example, the work of Mary Putnam Jacobi in the 19th century ^20^ (a doctor, scientist and suffragette) can be viewed as enhancing women’s status in society while trivializing or ignoring what for some women are very impactful biological processes. In any case, arguing for different treatment for women -- or any kind of person -- runs up against cultural ideas of fairness in which the slippage between equity and equality can create discord and resentment, and therefore this distinction deserves careful attention.

### *19 ^th^ Century psychiatry*: A woman's energy is needed for proper development of her female organs

**Figure 1.**
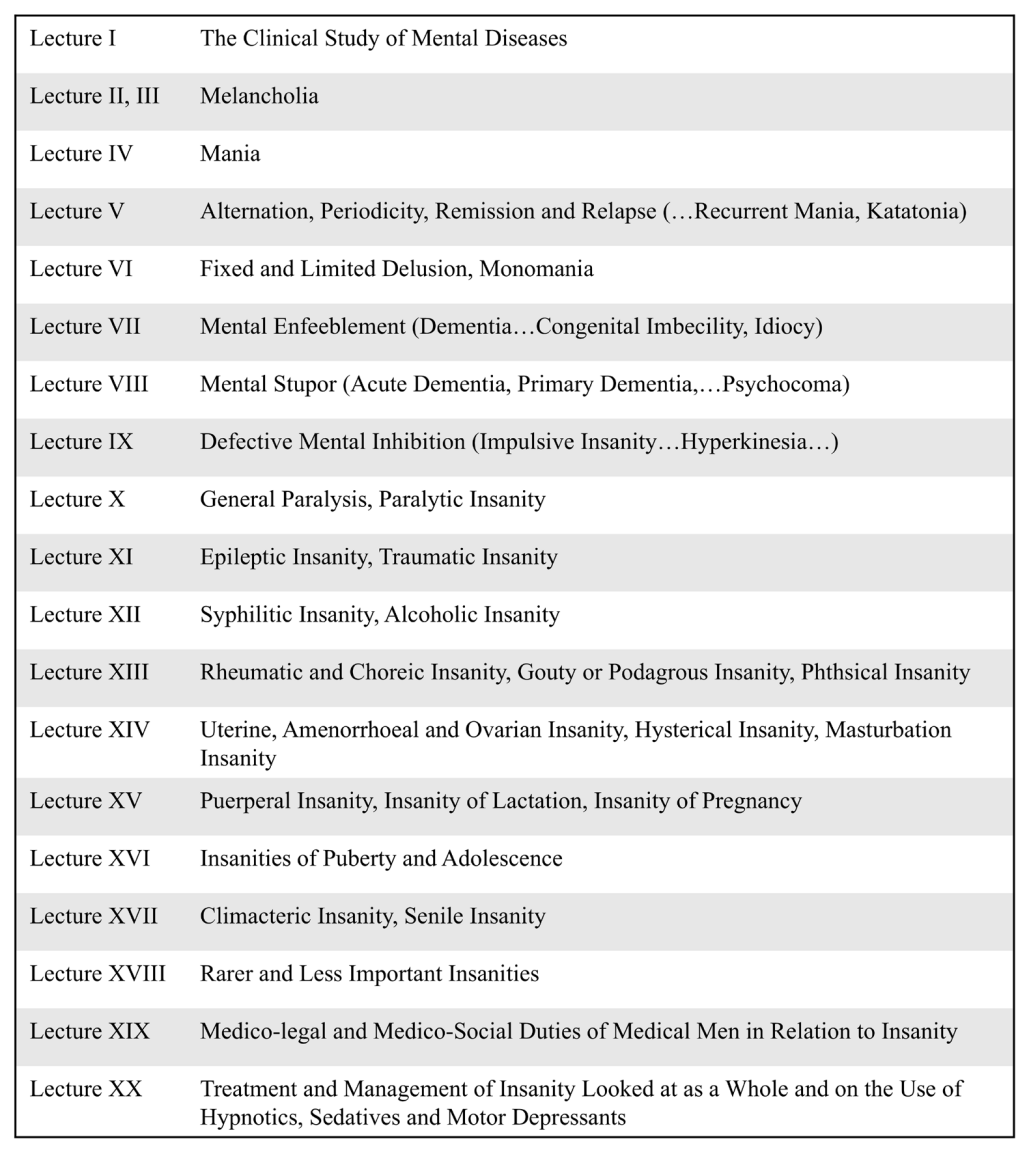
Abridged chapter titles of T.S. Clouston’s 1896 *Clinical Lectures on Mental Diseases* ^26^. Dr Thomas Clouston was Medical Superintendent of the Royal Edinburgh Asylum and the first official Lecturer in Mental Diseases at the University of Edinburgh in 1879. His *Clinical Lectures on Mental Diseases* was the recognised textbook for the students of Edinburgh’s Medical School, which was at this time at the height of its prestige as an internationally leading centre for the study of medicine. Clouston’s work was widely disseminated and alongside influential figures such as Henry Maudsley and Edward Clarke, was an authoritative voice in the field ^24^.

The perceived relationship between ‘madness’ and the female body, particularly the notion that women’s reproductive systems were the seat of a wide range of mental and physical disorders, is one that has captured the popular imagination and interest of Western scholars for centuries, if not millennia. The “hormonal female” is a tenacious trope, despite the fact that both male and female bodies produce oestrogen, progesterone and testosterone, plus rely on fluctuation of other hormones such as insulin and thyroid hormone to adaptively regulate their physiology on a daily basis. The nineteenth century, however, was a period notable for the resurgence of debates around the fragility of the female constitution, most notably amongst members of a new and growing medical specialty: psychiatry. The period 1870 to 1890, in particular, witnessed the proliferation of psychiatric literature that associated disorders of the mind with key transitions in the female life cycle ^21– 24^. A number of influential textbooks and clinical lectures devoted considerable space to the potential risks these crises of transition posed to the ‘fragile’ or ‘pathological’ female body, such as the internationally recognised *Clinical Lectures* of prominent nineteenth century Scottish psychiatrist Dr Thomas Clouston, in which the ‘body, lifecycle, and menstrual cycle constitute…something akin to leitmotifs,’ or recurring themes ^24^. It is notable that three of his seventeen chapters on the totality of mental diseases made reference to female reproductive events ( Figure 1).

Indeed, while both sexes were deemed to be at risk of mental instability during lifecycle ‘crises of transition’, it was stressed that periods such as puberty and adolescence were of particular danger to the inherently ‘fragile’ constitutions of women who were, in turn, more vulnerable to forms of distinctly ‘female’ insanity e.g. ovarian insanity, uterine insanity, amenorrhoeal insanity. It is particularly striking that the points at which menstruation begins, is disrupted or ceases entirely were focal points in this psychiatric literature, which stressed that the ‘regular normal performance of the reproductive functions is of the highest importance to the mental soundness of the female’ ^25^. Menstrual problems such as painful or heavy periods and disrupted or absent menstruation were recorded as both a cause and a symptom of insanity in both patient records and published medical literature, though as Clouston somewhat paradoxically noted in his *Clinical Lectures,* even ‘normal’ menstruation posed a risk to a woman’s mental state:

Disturbed menstruation is a constant danger to the mental stability of some women; nay, the occurrence of normal menstruation is attended with some risk in many unstable brains. The actual outbreak of mental disease, or its worst paroxysms, is coincident with the menstrual period in a very large number of women indeed ^25^.

This recognition of a connection between menstrual problems and women’s mental health had a key tension at its heart: menstruation was deemed a potentially pathological and even dangerous process, but it was also an important ‘normal’ function essential for women’s mental and physical health, and needed protecting.

As suggested by the following entry in the 1892 *Dictionary of Psychological Medicine*, the empirical observations of Clouston, and other prominent psychiatrists appear to have been accepted as a form of scientific progress, proof of the field’s contribution to an understanding of what were once complex ‘mysteries’ of the female body and mind:

The correlation of the sexual functions and nervous phenomena in the female are too common and too striking not to have attracted attention at all times; but it may confidently be affirmed, that it is only within quite recent years that we have had adequate knowledge to enable us to discuss the problems arising out of these relations with scientific precision ^27^.

This increased recognition of the impact of the menstrual cycle upon women’s mental health by contemporary psychiatrists may well have ‘enlarged understanding’ that ‘resulted later in further therapeutic advances’ ^28^. However, as Anne Digby rightly observes, there was also a ‘darker side to this process’ ^28^. The greater attention paid to the menstrual cycle and its points of transition in late nineteenth century texts was not necessarily accompanied by, or translated into, a progressive approach to women’s health. This is perhaps most clearly demonstrated by how psychiatric theories were used beyond the medical sphere to justify the exclusion of women from education. In the latter decades of the nineteenth century, psychiatric conceptualisations of the fragile menstruating body increasingly became entangled within wider contemporary public debates in Scotland, England, and the United States about the impact of secondary and higher education on women’s physical and mental health ^21, 24^.

In the arguments of influential psychiatrists and physicians of the time, over-study was deemed not only a strain on the supposedly weaker female constitution in general, but especially damaging during puberty and adolescence, a period in which young girls’ bodies were thought to be perilously unstable. Such arguments were underpinned by the idea that all organisms have a finite amount of vital energy available for different physical and psychological processes: if energy was directed at one process, it became unavailable for another. According to Clouston, adolescence was a period in which the vital energy was of utmost importance, particularly for young girls, for whom it was called upon in great quantities in order ‘to bring to the harmonious perfect of full womanhood these combined bodily and mental qualities’ ^29^. Education would monopolize all the available vital energy, misdirecting it from the womb to the brain, which according to Clouston could have disastrous consequences: ‘growth is stopped, the blood is thinned, the cheeks are pallid, the fat destroyed, the wondrous forces and faculties.... are arrested before they attain completion.... the damage is irreparable’ ^29^. Young girls were considered at risk of growing up weak in body and weaker in mind, a view that was shared by several of Clouston’s contemporaries, including renowned British psychiatrist Henry Maudsley ^30^.

That the ‘sex in education’ debates of the late nineteenth century were infused with psychiatric theories is suggestive of how increased recognition of women’s physical and mental health differences can be weaponised against them. The debate arose at a time when campaigns for women’s suffrage and entry to medical school began. Digby argues that the medical profession created a ‘man-made biological straightjacket’ that transformed supposedly ‘natural laws’ into ‘social conventions that reinforced restrictive gender roles’ ^28^. This is further supported by the discernible gap between the rhetoric of psychiatric literature, which devoted an increasing proportion of text to descriptions of these female diseases and thereby conveyed an impression of their importance, and information on their actual incidence, which was lacking ^24^. In-depth studies of asylum case notes have shown that only a minority of women were admitted to asylums because of a ‘perceived link’ between their reproductive systems and their illness ^21^. Even Clouston himself appears to have been aware that his arguments were based on subjective expertise rather than in measurable quantitative evidence: ‘the weak point of my argument is that it is not founded on any basis of collated statistical facts’ ^31^. This gap between rhetoric and reality may partly explain why the views of prominent psychiatrists, though widely endorsed by a predominately male medical profession, were subject to greater criticism by female physicians including Mary Putnam Jacobi, whose in depth study 'The Question of Rest in Menstruation' ^20^ used quantifiable evidence to point to the contradiction between dominant theories and practice.

This gap between rhetoric and reality may partly explain why the views of prominent psychiatrists, though widely endorsed by a predominately male medical profession, were subject to greater criticism in the public sphere, where they proved far more divisive ^24^. Indeed, by the early decades of the twentieth century, ideas around the fundamentally pathological menstruating body were increasingly challenged and became untenable alongside the emergence of the ‘new woman’, protests for women’s rights, and the increasing numbers of women entering employment outside the home, particularly during the First World War ^22^. A more positive language of menstrual ability was promoted, including by the medical profession, one that emphasized menstruation as a ‘natural function’ rather than an illness ^22^. Yet, as the following sections will demonstrate, the paradoxes and tensions that accompanied the medicalisation of menstruation were never fully resolved but rather reconfigured, repackaged and expressed in new ways.

### *20 ^th^ century hormone replacement therapy (HRT):* “Once the ovaries stop, the very essence of being a woman stops”

The modern menopause ‘groans under centuries of cultural baggage’, with descriptions in medical literature linking it to numerous unpleasant conditions including death as far back as 16 ^th^ century Venice ^32^. Although the menopause looks very different across different times, places, and social groups, including its biological manifestations, negativity around the menopause is long-standing in Europe and North America ^33^. In contexts where women’s aging is less stigmatised and associated with decline, menopause symptoms are milder ^34^, although the relative contribution of factors such as genetics, diet and culture are unclear. In the mid 19 ^th^ century, an American physician, William Tyler Smith, ‘discovered’ menopausal hot flushes as a ‘disease’ linked to mental disorder:

The so-called “heats and chills” of this period consist of a real paroxysmal affection, allied in its nature both to intermittent fever and epilepsy… In fact this malady is a fruitful source of mania, occurring in the female after the decline of the catamenia ^35^.

**Figure 2.**
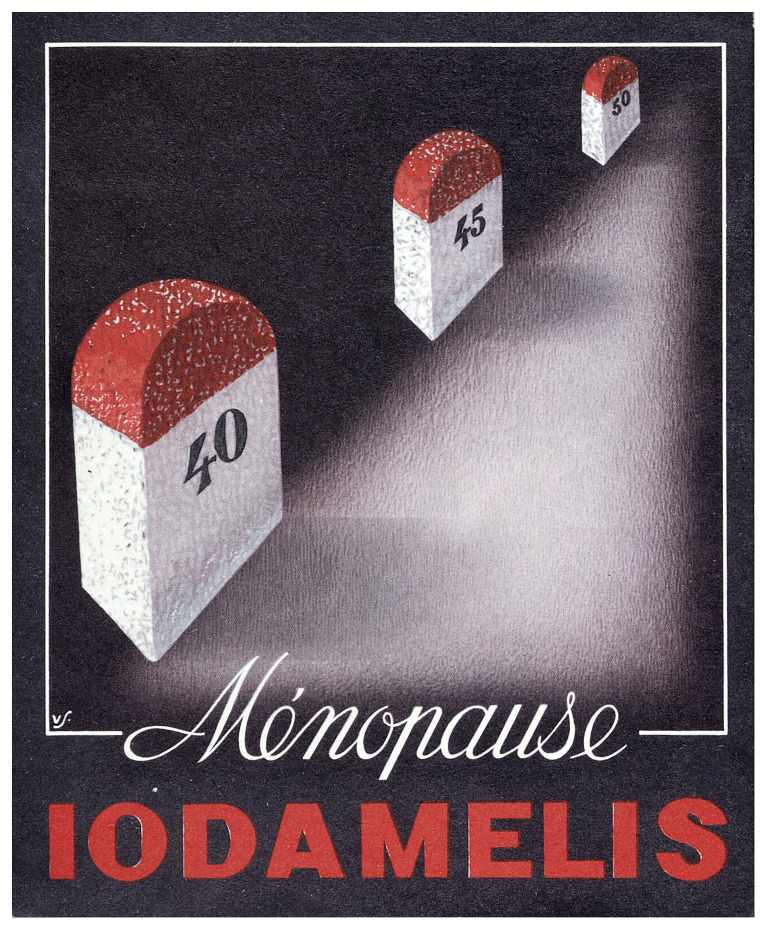
Postcard sent to physicians in France by a pharmaceutical company between the 1940s and 1960s advertising the use of Iodamelis (iodine) for treatment of menopausal symptoms, “looking like nothing so much as a series of tombstones stretching into a dark and forbidden future” ^36^. Source: Wellcome Collection 11667i, made available under a Creative Commons Attribution 4.0 International License.

When such vasomotor symptoms were first recognized in medical literature, treatments included the profoundly unpleasant-sounding application of leeches to the labia and cervix and iced water into the vagina and rectum ^35^. By contrast, the 20 ^th^ century proposition of pharmacological agents to alleviate menopausal symptoms appears considerably more humane, dignified, and scientific – yet comes with a complex set of trade-offs. Notable among them is how corporations reiterate and enforce the association of the menopause with ageing and death to serve their own interests ( Figure 2).

Hormone replacement therapy became popular in the 1960s. A bestselling book written by the gynecologist Robert A. Wilson, *Feminine Forever,* framed estrogen as a magical substance that could make women ‘once again’ attractive and sexually available, rendering the menopausal woman “much more pleasant to live with” ^37^. The New York Times later reported that Wilson was paid by the manufacturer of Premarin, the most popular estrogen treatment throughout the 20 ^th^ century ^38^. The message landed: by 2001, 42% of women aged 50–74 living in the USA had been exposed to HRT ^39^. In the same era, the psychiatrist David Reuben wrote that “Once the ovaries stop, the very essence of being a woman stops” in his book *Everything You Always Wanted to Know About Sex but Were Afraid to Ask,* adding that the postmenopausal woman comes “as close as she can to being a man” -- or rather, “not really a man but no longer a functional woman” ^40^. It is striking that this was only 50 years ago. The misogyny of these statements is obvious today – but the changing marketing of menopause medicine still rests on a core of, at best, ambivalence about older women.

**Figure 3.**
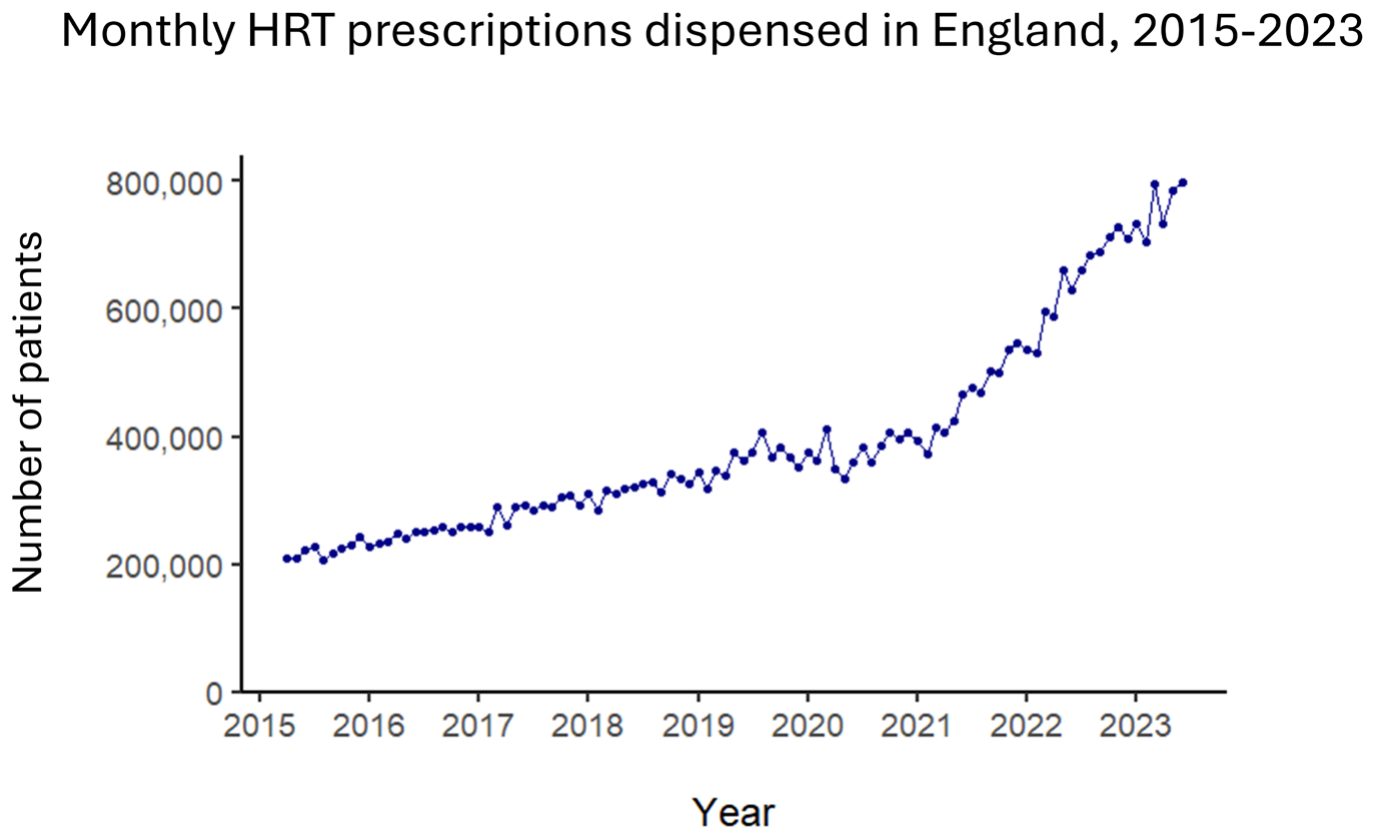
HRT prescriptions dispensed in community pharmacies each month in England, April 2015 - June 2023. Number of patients refers to identified patients. Source: the data plotted are publicly available from NHS Business Services Authority data repository at https://www.nhsbsa.nhs.uk/statistical-collections/hormone-replacement-therapy-england/hormone-replacement-therapy-england-april-2015-june-2023

Today, hormonal treatments for the menopause are at the centre of an increasingly polarised debate. While HRT prescriptions dropped precipitously after the 2002 Women’s Health Initiative study linking them with breast cancer and stroke ^41^, subsequent re-analysis of this data stratified by age, and new evidence in younger women, has instead found reductions in coronary heart disease and all-cause mortality in women where HRT is started within ten years of the menopause ^42^. There has been an upward trend in prescriptions since 2015 which is becoming increasingly steep ( Figure 3), with expanded production facilities opening in Europe in 2023 ^43^. The same year, a New York Times Magazine piece elaborated the perspective that denying women HRT is a misogynistic abuse that turns a blind eye to women’s suffering ^38^. Yet others, such as gynaecologist Martha Hickey, react against an “over-medicalisation” of the menopause that serves commercial companies and individuals with vested interests, arguing that it should be viewed primarily as a natural transition ^44^.

The current British Menopause Society and NICE guidelines recommend a holistic approach including lifestyle advice and offering HRT to patients experiencing menopausal symptoms (vasomotor symptoms, mood symptoms, sexual function, urogenital atrophy), but do not recommend HRT for those who are perimenopausal but asymptomatic (unless indicated for other reasons e.g., premature menopause, osteoporosis prevention) ^45, 46^.

Here, we are not taking a stance in this debate. Our aim here is to show how developments in “hormonal thinking” in the 20 ^th^ century led to this polarized situation, which can help us understand how medicalising the menopause has both good and bad effects. In the 1930s, pharmaceutical oestrogen was becoming available while oestrogen was becoming associated with womanhood and, non-coincidentally, the menopause was becoming understood as hormonal deficiency, as historian Nelly Oudshoorn explains ^47^. The fact that women’s aging was medicalised in this way while men’s was not (despite the potential profitability of, for example, testosterone supplements to “increase virility”) was influenced by the material conditions of hormone science. While clinical gynaecology made it easy to collect hormone-containing tissues and market drugs to women, human testes and male urine were very difficult to obtain – the medical practice of andrology did not exist until the 1960s, and even now is obscure. So, despite attempts in this era to classify symptoms of aging men as ‘male menopause,’ dirnacterium, or andropause, the organizational infrastructure of medical institutions alongside men’s attitudes towards health problems were not conducive to medicalizing men’s bodies (a kind of institutional ‘looping effect’). 

Framing this period of transition as a disease of hormonal deficiency that can (and should) be eased only by replacing the missing hormones is reductive. Of course women should have access to treatments that help them feel well. But reducing this complex socio-biological life transition to a “deficiency” writes out all the social factors and misogyny that make aging as a woman more challenging than aging as a man, in many ways exacerbating stigma and fueling negative attitudes to the menopause. It erases the quarter of women who have no symptoms and the two thirds of women whose symptoms are mild ^45^, leaving no room for the menopause to be a non-issue or a differently-experienced issue. And, although it is more easily visible in the blatantly sexist language from the 1960s, above, the normative aspects of this hormone-deficiency framing perpetuate a very blurry boundary between “feeling good” and “living up to social expectations of womanhood.”

If we draw the idea that HRT is needed for everyone to its logical extreme, women should never stop having periods, and indeed deliberately extending women’s reproductive lifespan using rapamycin is currently under assessment in a clinical trial ^48^. The question of what is “natural” looms large here – yes, the menopause is “natural” but “One could argue very reasonably that most chronic disease is natural, childbirth is natural, death is indeed natural, declining mental function is natural, declining eyesight and hearing are natural but the medical profession and healthcare workers aim to improve all of these life events or problems without question” ^49^. The question then becomes what it would look like to age *well*, and who gets to decide. Middle or third age female celebrities are becoming CEOs of menopause related companies ^50^, which have an aggressively positive spin on the benefits of participating in their medicalisation and the rejection of aging (‘girl boss feminism’ within male-dominated structures). If we look for an analog to HRT in Viagra and other sex-enhancing drugs targeting older men, we might 1) note their ubiquity and general lack of shame in marketing, but moreover 2) consider examples of men rejecting such drugs and the medicalisation of aging in order to age respectably ^51^. We might also consider trans and gender affirming healthcare as a model that begins from the premise of asking “what do you want your body to be like and do for you” rather than a culturally defined idea of what people of any gender should expect and want as they age.

### *21 ^st^ century free period products and menstrual leave:* "A woman needs special help"

In 2021, Scotland passed an historic Period Products Act, making it the first country in the world to legislate universal access to free period products. It was internationally heralded as groundbreaking, though issues with enforcement and follow-through are ongoing. Along with the ways in which it was indeed novel and part of a wider contemporary ‘menstrual moment’, it continues some traditional conceptions of menstruation (such as a stigma that should remain hidden, and a problem to be solved with industrial products) ^52^. Also recently, Spain trialled menstrual leave policies allowing people to take time off work for reasons related to their periods. It has likewise received attention for the novelty of this, although actually menstrual leave policies originated in Russia in 1880 and post-World War II East Asia as a pronatalist strategy (before timing of implantation was understood) ^53^. Both well-intentioned policies are at the cutting edge of menstrual equity activism ^54^, entwined with the growing role of social media platforms in raising awareness around menstrual health, period poverty and advocacy for policy changes (e.g. 55). And, both policies are accompanied by the risks of medicalising menstruation.

Of course, provision of free menstrual products helps those experiencing poverty or unpredictable cycles or who simply forgot or would prefer to spend their money on something else. This is a welcome development for how significantly it can reduce the stress, shame, and negative emotions of people who menstruate (lack of access to basic needs has significant mental and social consequences) ^56^. Of particular note here is access to period products in institutional settings where vulnerable people find themselves, such as psychiatric wards and prisons. The continued impact of menstrual stigma means that many menstruators still feel embarrassed about having to speak openly about their need for products, or to justify this need through details on their menstrual experience ^57^. If perfectly executed, it would be equitable in that no one must carry around supplies to maintain their own hygiene (no one is expected to carry around their own toilet paper in the UK or other wealthy countries). Yet it is not *equal* if women are seen as requiring special treatment, which can generate some backlash. If period poverty – the inability to pay for menstrual products -- is the problem, then providing them for free is in some sense a stop-gap for the broader problem of poverty. Ensuring that everyone can afford all household essentials would be a more gender-liberatory aim. There are, of course, reasons that this more ambitious agenda is more challenging (one of the reasons the Period Products Act was successful is its relatively low cost to the state ^52^), but it bears noticing that addressing *period poverty* in particular makes periods a problem and public health issue, and menstruating people a burden on society. In an alternative scenario, for example, pads could be provided in *all* toilets with unforeseen benefits: anecdotally, when a start-up company producing rolls of tear-off pads to be installed next to toilet paper rolls trialled them in football stadiums, older men with incontinence made even more use of the pads than women attending the games (personal communication during an anniversary celebration of the Period Products Act at the Scottish Parliament).

Leave policies are an intriguing way to recognize that some menstruating people’s abilities shift dramatically while on their period, whether due to pain, heavy flow, energy levels, or other experiences. In the last 25 years, Zambia, Taiwan, two industrial regions of China and a handful of corporations have introduced menstrual leave ^53^. In such policies’ recent iteration, they have been proposed with the goal of reducing suffering and making workplaces more accommodating, but they introduce troubling (and precedented, as we have seen!) parallels between womanhood and impairment. In their gendered form, such policies have been shown to increase gender-based resentment and discrimination, including lower recruitment and retention of women in multiple countries where menstrual leave has been implemented ^58^. A survey of 600 people in the USA, 315 of whom were men, about attitudes towards menstrual leave found that 21% felt the policy would be unfair to men ^59^. One respondent stated “…while it sucks men show up to work a lot of times even if they are sick, in pain, half bleeding” ^59^. Across the whole of Spain, only around 5 people a day took up the opportunity for this leave within the first year of the legislation being enacted, with the need to request leave via a doctor mentioned as one of the reasons for low uptake ^60^, which may relate to stigma and/or the practical burden of obtaining a doctor’s appointment.

If workplaces have sufficiently comprehensive employee rights, then special menstrual leave is not needed (an analysis supported by the fact that historically, menstrual leave policies have arisen in industries where there are inadequate rest or toilet breaks such as factories and sweat shops) ^53^. As King summarises:

By framing the problem as one of menstrual health, rather than poor or inadequate working conditions, menstrual leave policies reinforce inaccurate beliefs used to justify gender inequality by positioning the female body as abnormal, inferior or disproportionately prone to illness ^61^.

Such policies are welcome to the extent that they make way for policies and cultural expectations that recognize that *most* people (of all genders) have fluctuating energy levels, discomforts, and abilities. Menstrual leave also perpetuates the idea that common or healthy menstruation is painful or debilitating, normalising such ‘symptoms’ and potentially advancing the idea of ‘all women’ as inherently more complicated, weak, unreliable, or expensive employees ^61^. Debilitating pain is not the norm (experienced by 2–29% of women ^62^), and rather than sending people who experience it home, out of the public sphere, wouldn’t it be preferable to support them to receive treatment? There is a defeatist attitude inherent in the policy, suggesting that women should just endure. Menstruation-friendly workplaces need to account not only for period products and leave but different options for time away from work (including to use the washroom as needed), toileting and washing spaces, harassment, education, and various possible indignities ^54^; they need to be co-designed with the people they aim to serve ^63^.

A larger issue across both these policies is how difference (or perceived difference) aligns with social expectations. What about menstruation (or about bodies) is society's problem versus a personal problem? Anthropologist Emily Martin’s classic study of the medicalisation of women’s bodies (first published in 1987 and reissued in 2008) provides interesting perspective on this ^64^. She traces a 20 ^th^ century history of PMS, which was claimed as a reason women couldn’t work following both World Wars when jobs ‘needed’ to be returned to men, though suppressed as a reason during those wars (for example, Rosie the Riveter campaigns). Institutional and cultural concern with PMS rose again in the 1970s after birth control use became widespread and women were pursuing careers before or in lieu of childbearing. Martin describes this as "The conjunction between periods of recent history when women's participation in the labour force was seen as a threat, and simultaneously, menstruation was seen as a liability ^64^." Does the gradual rise in women in senior positions since Martin published her piece now subconsciously drive the desire to place them on menopause leave at a time of power or promotion?

It will be clear by now that drawing attention to (perceived) fluctuations in women’s abilities has its discriminatory downsides; yet there are those who take as a starting point that menstruation and cyclicity *does* impact one’s aptitudes and claim that difference is not an impairment but a superpower. Contemporary “cycle syncing” urges people to align their expectations and activities with ideas about fluctuations (commonly, high energy and sociability during ovulation, and retreating energy during the luteal phase: the “hormonal roller coaster”), asserting that this will streamline their life and optimize their capacities. A burgeoning science of menstruation and sports training, for example, attempts to use menstruators’ cyclicity to maximize their athletic capacity. People do find this helpful (particularly those already accustomed to disciplining their lives in order to attain educational and professional success), but it also promotes an exhausting imperative to optimise and capitalise upon every experience ^65, 66^. Martin anticipated the appeal of these discourses, wondering whether symptoms of PMS are intrinsically problematic or whether modern work practices and societal expectations make them so? In her prescient words, "Women are perceived as malfunctioning and their hormones out of balance rather than the organisation of society and work perceived as in need of a transformation." And yet, cycle syncing and products and services promoting ‘hormonal balance’ put this pressure back on individuals, instead of changing social expectations (for everyone, not just women).

## Conclusions

**Figure 4.**
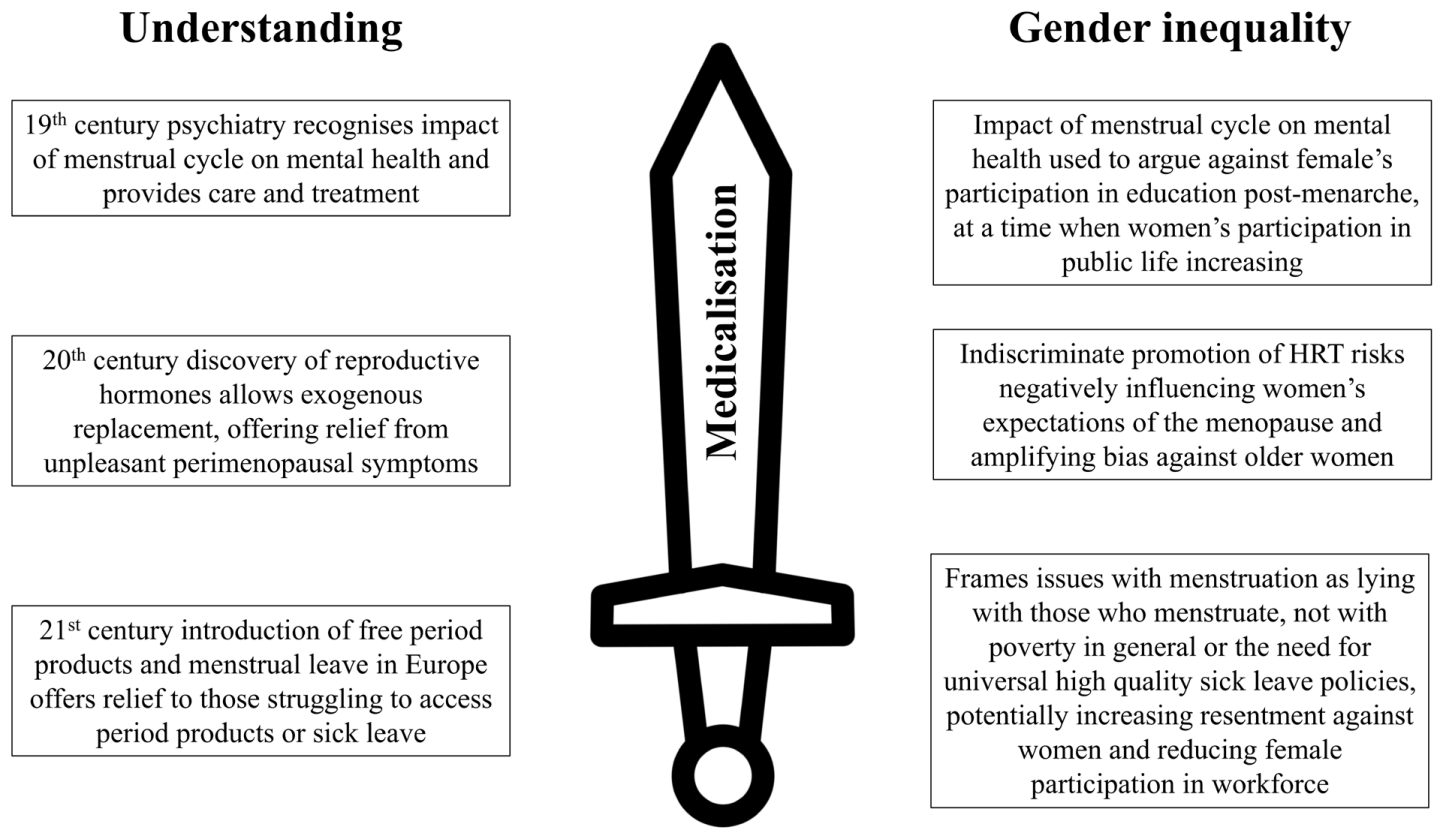
The medicalisation of menstruation as a double-edged sword.

**Figure 5.**
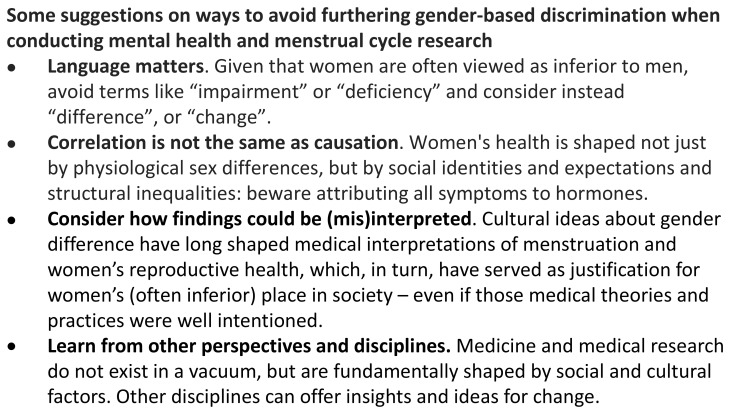
Avoiding furthering gender-based discrimination when conducting female reproductive health research.

Any changes come with desirable and undesirable possibilities (and what is desirable and undesirable varies with one’s perspective) ( Figure 4). We do not take a prescriptive stance on what would be good, or what should happen, but encourage practitioners and researchers to have a bit of wariness and a critical eye when marching towards “solutions.” We hope the material reviewed here will help in learning from past mistakes and encourage thinking across disciplines, and using creativity and imagination about how best to innovate. We have made some practical suggestions to reflect upon ( Figure 5). We have tried to draw attention to the weight of history, culture and society, how they shape how we draw categories and with what effect. Biological experiences are shaped by cultural attitudes toward the body, and all categories are value-laden – biological facts are not fully objective truths: it’s not so straightforward. While medical theories/conceptualizations of menstruation have been in constant flux, oscillating between the pathological and dangerous on one end of the spectrum, and the normal, healthy and even essential, on the other, what is clear is that the female body continues to be a site for the definition and construction of the female as “other” ^22^, even in the twenty first century, despite the good-intentions of medical practitioners.

## Ethic and consent

Ethical approval and consent were not required.

## Data Availability

No data are associated with this article.

## Authors’ information

Dr Andrea Ford is a medical anthropologist. Dr Jessica Campbell is a medical historian. Dr Katie Marwick is a psychiatrist.
