# Supplementary material for: The medicalisation of menstruation: a double-edged sword
Source: Wellcome Open Res. 2025 Sep 26;10:204. Originally published 2025 Apr 23. [Version 2] doi: 10.12688/wellcomeopenres.24017.2 (PMC12553977; doi:10.12688/wellcomeopenres.24017.2)
Supplement: Peer review history

# Reviewer report: Reviewer response for version 2

**DOI:** 10.21956/wellcomeopenres.27566.r134451

**Published:** 2025-10-25

**Authors:** Lara Owen

**Affiliations:** 1 University of St Andrews, St Andrews, Scotland, UK

I have now read the author notes and the revised paper. I appreciate the changes made and I have no further comments.

Is the review written in accessible language?

Yes

Are all factual statements correct and adequately supported by citations?

Yes

Are the conclusions drawn appropriate in the context of the current research literature?

Partly

Is the topic of the review discussed comprehensively in the context of the current literature?

Partly

Reviewer Expertise:

Culture, politics and organisation of menstruation and menopause. Menstrual stigma including its history. Menstruation in the workplace.

I confirm that I have read this submission and believe that I have an appropriate level of expertise to confirm that it is of an acceptable scientific standard.

# Reviewer report: Reviewer response for version 2

**DOI:** 10.21956/wellcomeopenres.27566.r134450

**Published:** 2025-10-08

**Authors:** Lotte Gerritsen

**Affiliations:** 1 Utrecht University, Rotterdam, The Netherlands

Based on the summary of the revision I would recommend to accept this manuscript for indexing.

Is the review written in accessible language?

Yes

Are all factual statements correct and adequately supported by citations?

Yes

Are the conclusions drawn appropriate in the context of the current research literature?

Yes

Is the topic of the review discussed comprehensively in the context of the current literature?

Yes

Reviewer Expertise:

Neuroscience, Clinical Psychology, Female mental health, Hormones, Aging and Epidemiology

I confirm that I have read this submission and believe that I have an appropriate level of expertise to confirm that it is of an acceptable scientific standard.

# Reviewer report: Reviewer response for version 1

**DOI:** 10.21956/wellcomeopenres.26497.r126463

**Published:** 2025-08-29

**Authors:** Lara Owen

**Affiliations:** 1 University of St Andrews, St Andrews, Scotland, UK

**Overview**

 I like the pulling together of similar themes across disciplines, contexts, and historical periods. Broadly speaking, this paper is a useful reminder, from various points in history, of ways in which even well-meaning attention placed on menstruation can be employed to entrench misogyny rather than to correct it.

 While I appreciate this paper is an overview and that therefore opportunities for detail are limited, I do feel the piece would benefit from a little more detail on ways in which stigma is reproduced. I have given some examples of this in my comments below that will not unduly elongate the text or introduce new themes.

 In terms of the conclusion, perhaps the addition of the concepts of imagination and innovation would help the paper to not feel quite so gloomy. I am sure you are not intending to come across as saying that any attempts to make menstruating experience better are doomed, but you could suggest more assertively that we need to think better and with more ingenuity, and not to fall for traps that research has already shown to be present (e.g. the Spanish bill was clearly going to fail, from research data that we already had by then).

**Comments and suggestions**

 (Quotes in italics, comments in regular text.)

 Introduction paragraph 4, last sentence.

*Further, a randomised controlled trial that found that treating PMS with CBT delivered to couples was more effective in some domains than CBT delivered to the affected individual only*. This sentence could use an explanatory clause such as "indicating that relationship and/or family dynamics, such as inequity in domestic chores and childcare, may be the root cause of the apparent PMS". See e.g. Ussher and Perez 2012.

 Intro para 6

*An updated version might propose individual non-gendered toilets as a solution that is both equal and equitable (with enclosed sinks allowing for hygiene needs of various sorts to be addressed privately). *This solution neglects to consider the different ways that genders and classes are socialised to behave in toilets. Women (and cleaners, who are usually women) report that men routinely leave more mess (urine splashes, faeces streaks, toilet paper on the floor etc) than women tend to do, in domestic as well as public toilets. At the same time, women are socialised to clean up after men, while men are more likely to feel insouciant about leaving mess behind them. So perhaps this solution is not as equal or equitable as it might at first seem.

 HRT section, para 5, is unclear on what within it is a quote if anything, and this lack is particularly pertinent for the last sentence. Who is "we" here?: *Of note for practicing psychiatrists are the current British Menopause Society and NICE guidelines which recommend a holistic approach including lifestyle advice and offering HRT to patients experiencing menopausal symptoms (vasomotor symptoms, mood symptoms, sexual function, urogenital atrophy), but do not recommend HRT for those who are perimenopausal but asymptomatic (unless indicated for other reasons e.g., premature menopause, osteoporosis prevention) ^42, 43^. **We can help our patients** navigate this increasingly fraught area through listening, informing and shared decision making.*

 Period products and policies section:

*It is degrading to have to explain what you need: *an odd shift in tone and reads more like an opinion piece than an academic article. Why the second person? Why insert an assertion without context? Instead you could say "The continued impact of menstrual stigma means that many menstruators still feel embarrassed about having to speak openly about their need for products, or to justify this need through details on their menstrual experience". Similarly, any requirement to prove the existence of validated menstrual symptoms, such as a medical note, can be a barrier to uptake of menstrual workplace policies (see next para, where you could expand on the last sentence by putting it in the context of menstrual silencing due to stigma).

*If period poverty – the inability to pay for menstrual products -- is the problem, why not address poverty in general and ensure that everyone can afford all household essentials? * This is a rather spurious argument. Why do anything?

*Anthropologist Emily Martin’s 2008 classic study of the medicalisation of women’s bodies (originally published 1987) -- *even if you are referencing the 2008 edition, this is still clunky and could be confusing to the reader. This book is always referred to as a work of 1987, and the date is important and is why it is characterised as a classic study. Better to write: "Anthropologist Emily Martin’s classic study of the medicalisation of women’s bodies (first published in 1987 and reissued in 2008) provides interesting perspective on this ^61^. She Martin traces a 20 ^th^ century history of PMS..."

Is the review written in accessible language?

Yes

Are all factual statements correct and adequately supported by citations?

Yes

Are the conclusions drawn appropriate in the context of the current research literature?

Partly

Is the topic of the review discussed comprehensively in the context of the current literature?

Partly

Reviewer Expertise:

Culture, politics and organisation of menstruation and menopause. Menstrual stigma including its history. Menstruation in the workplace.

I confirm that I have read this submission and believe that I have an appropriate level of expertise to confirm that it is of an acceptable scientific standard, however I have significant reservations, as outlined above.

# Response

**Published:** 2025-09-23

**Authors:** Katie Marwick

**Affiliations:** University of Edinburgh, UK

We thank the reviewer for their positive comments on the paper and for their helpful suggestions for improvement. We have incorporated these as below. We now provide a point by point response:

 1. In terms of the conclusion, perhaps the addition of the concepts of imagination and innovation would help the paper to not feel quite so gloomy. I am sure you are not intending to come across as saying that any attempts to make menstruating experience better are doomed, but you could suggest more assertively that we need to think better and with more ingenuity, and not to fall for traps that research has already shown to be present (e.g. the Spanish bill was clearly going to fail, from research data that we already had by then). We have incorporated a sentence to this effect in the conclusion: “We hope the material reviewed here will help in learning from past mistakes and encourage thinking across disciplines, and using creativity and imagination about how best to innovate.”  

 2. Introduction paragraph 4, last sentence.

*Further, a randomised controlled trial that found that treating PMS with CBT delivered to couples was more effective in some domains than CBT delivered to the affected individual only*. This sentence could use an explanatory clause such as "indicating that relationship and/or family dynamics, such as inequity in domestic chores and childcare, may be the root cause of the apparent PMS". See e.g. Ussher and Perez 2012. We have added a clause and reference for further reading as follows “suggesting that relationship dynamics may at least in part contribute to PMS symptoms [Ussher and Perz 2010].”  

 3. Intro para 6

*An updated version might propose individual non-gendered toilets as a solution that is both equal and equitable (with enclosed sinks allowing for hygiene needs of various sorts to be addressed privately). *This solution neglects to consider the different ways that genders and classes are socialised to behave in toilets. Women (and cleaners, who are usually women) report that men routinely leave more mess (urine splashes, faeces streaks, toilet paper on the floor etc) than women tend to do, in domestic as well as public toilets. At the same time, women are socialised to clean up after men, while men are more likely to feel insouciant about leaving mess behind them. So perhaps this solution is not as equal or equitable as it might at first seem.

   Thank you for highlighting this concern, we have added a sentence and reference to this effect:  “However, gender-neutral toilets are sometimes viewed as less clean, related to different genders’ socialization around tidiness [Lewkowitz and Gilliland 2025].“  

 4. HRT section, para 5, is unclear on what within it is a quote if anything, and this lack is particularly pertinent for the last sentence. Who is "we" here?: *Of note for practicing psychiatrists are the current British Menopause Society and NICE guidelines which recommend a holistic approach including lifestyle advice and offering HRT to patients experiencing menopausal symptoms (vasomotor symptoms, mood symptoms, sexual function, urogenital atrophy), but do not recommend HRT for those who are perimenopausal but asymptomatic (unless indicated for other reasons e.g., premature menopause, osteoporosis prevention)* *^42^* *^,^* *^43^* *. **We can help our patients** navigate this increasingly fraught area through listening, informing and shared decision making.*

   There are no quotes in this paragraph, it is a summary of guideline statements. We apologise for the out of place use of “we” and have deleted this sentence. We have also deleted the mention of psychiatrists, as health care practitioners and psychiatrists are only one potential audience for this manuscript.  

 5. Period products and policies section:

*It is degrading to have to explain what you need: *an odd shift in tone and reads more like an opinion piece than an academic article. Why the second person? Why insert an assertion without context? Instead you could say "The continued impact of menstrual stigma means that many menstruators still feel embarrassed about having to speak openly about their need for products, or to justify this need through details on their menstrual experience". Similarly, any requirement to prove the existence of validated menstrual symptoms, such as a medical note, can be a barrier to uptake of menstrual workplace policies (see next para, where you could expand on the last sentence by putting it in the context of menstrual silencing due to stigma).

   Thank you for this suggested improved statement, which we have gratefully incorporated. We have also added a clause to the end of the menstrual leave paragraph “…., which may relate to stigma and/or the practical burden of obtaining a doctor’s appointment.”  

*6. If period poverty – the inability to pay for menstrual products -- is the problem, why not address poverty in general and ensure that everyone can afford all household essentials? * This is a rather spurious argument. Why do anything? We have rephrased this point as: “If period poverty – the inability to pay for menstrual products -- is the problem, then providing them for free is in some sense a stop-gap for the broader problem of poverty. Ensuring that everyone can afford all household essentials would be a more gender-liberatory aim.“

*7. Anthropologist Emily Martin’s 2008 classic study of the medicalisation of women’s bodies (originally published 1987) -- *even if you are referencing the 2008 edition, this is still clunky and could be confusing to the reader. This book is always referred to as a work of 1987, and the date is important and is why it is characterised as a classic study. Better to write: "Anthropologist Emily Martin’s classic study of the medicalisation of women’s bodies (first published in 1987 and reissued in 2008) provides interesting perspective on this ^61^ . She Martin traces a 20 ^th^ century history of PMS..." Thank for you this suggestion for improved clarity, which we have incorporated.

# Reviewer report: Reviewer response for version 1

**DOI:** 10.21956/wellcomeopenres.26497.r122706

**Published:** 2025-05-08

**Authors:** Lotte Gerritsen

**Affiliations:** 1 Utrecht University, Rotterdam, The Netherlands

This article by Ford et al. addresses a immensely important topic, as this is exactly what many researchers and clinicians are currently struggling with. Knowledge should be empowering, but indeed we already see that the increasing awareness and knowledge can be misused. On one hand female specific health aspects have been mostly ignored because the majority of researchers did not feel it was relevant, or because researchers were afraid by including women as participants their results would be biased (as men were considered the control condition). While on the other hand women purposefully ignored the female specific aspects, because they needed to show they were equal to men. These two historic processes resulted in women's health not being studied and understood at all. Fortunately, there is increased awareness of the need to increase our understanding of women specific health aspects, but we do not want to fall into same wrongful assumption of before that deemed 'women are weak because of their hormones'. As such this article provides the right tools to analyze and understand the double sworded problem of on one hand we applaud all attention to women specific health aspects but on the other hand we truly do not want these insights to be used against women/people who are menstruating/people with an uterus.

 I very much like that by using three very clear examples the case is made for their rethoric on the double-edged sword. I think it is also very good to start with the explanation of the used concepts.

 However there are a few aspects that I miss, which also play a role in this story:

 1) See the work by Mary Putnam Jacobi. She has written several books in the late 19th century to show that women are still able to function and do not need to rest or are frail during menstruation.

 At the same time what she and women from the first feministic movement did was totally downgrading women specific health aspects so that women would not be seen as weak. This is fully understandable given the unequal position women were in at that time.

 This however also increased the trivialisation of (benign) gynaecological disorders such as pelvic pain, endometriosis, PMDD, peri-natal health etc.  And up till today we are struggling against this view that all women specific aspects are not relevant, or are not real, or are natural and you should just deal with it. Whereas, some women (not all!) do however suffer enormously from women specific health aspects and definitely do need better care.

 2) All humans are hormonal. Somehow only women are thought to be hormonal, and because of that women have often been excluded from biomedical research, because it was believed that their hormonal cycles would influence the results. Also see the excellent work by Rubi et al 2023 in Current Biology. Now the point here is: yes their hormonal status may affect the pharmacokinetics, neurobiology, immunology etc. but that is exactly why we should study it, because otherwise we do not know how women are affected by drugs and illnesses. This is where we currently stand: the awareness that a female body is not just a smaller version of the male body with some additional parts, but that in some aspects it actually works differently.

 By including women we can also improve healthcare for men, because men are not the control condition, men and women need sex-specific health care. We are only at the start of this revolution in medicine.

 It would be good if these points could be addressed in some way, as I believe it would make the story more complete. I actually do not agree that we should change our language, but that we should show more how by increasing our understanding of women specific health aspects we will improve healthcare for all.

 Ideally in the future we can get rid of the idea that the term hormonal only refers to women.

 1) All animals are hormonal. All sexes have the same sort of hormones, but in different levels.

 2) Besides the sex hormones there are various hormones that play a role in appetite, sleep, energy levels, growth etc. 3) In all animals hormones fluctuate during the day, as the key role of hormones it to keep us balanced and well equipped to react to changes in the world around us.

 So yes we are all hormonal.

Is the review written in accessible language?

Yes

Are all factual statements correct and adequately supported by citations?

Yes

Are the conclusions drawn appropriate in the context of the current research literature?

Yes

Is the topic of the review discussed comprehensively in the context of the current literature?

Yes

Reviewer Expertise:

Neuroscience, Clinical Psychology, Female mental health, Hormones, Aging and Epidemiology

I confirm that I have read this submission and believe that I have an appropriate level of expertise to confirm that it is of an acceptable scientific standard, however I have significant reservations, as outlined above.

# Response

**Published:** 2025-09-23

**Authors:** Katie Marwick

**Affiliations:** University of Edinburgh, UK

We thank the reviewer for their positive comments on the overall aim and content of the article. We are grateful for their suggestions for improvement, which we have incorporated as below. We now provide a point by point response:

 1. However there are a few aspects that I miss, which also play a role in this story:

 1) See the work by Mary Putnam Jacobi. She has written several books in the late 19th century to show that women are still able to function and do not need to rest or are frail during menstruation.

 At the same time what she and women from the first feministic movement did was totally downgrading women specific health aspects so that women would not be seen as weak. This is fully understandable given the unequal position women were in at that time.

 This however also increased the trivialisation of (benign) gynaecological disorders such as pelvic pain, endometriosis, PMDD, peri-natal health etc.  And up till today we are struggling against this view that all women specific aspects are not relevant, or are not real, or are natural and you should just deal with it. Whereas, some women (not all!) do however suffer enormously from women specific health aspects and definitely do need better care. Thank you for bringing Professor Jacobi’s work to our attention. We have summarised this point as follows: “Attempts by women themselves to downplay biological differences from men, for example, the work of Mary Putnam Jacobi in the 19 ^th^ century [Jacobi 1876] (a doctor, scientist and suffragette) can be viewed as enhancing women’s status in society while trivializing or ignoring what for some women are very impactful biological processes. “ We have also added a mention of her work to the 19 ^th^ century section as follows: “This gap between rhetoric and reality may partly explain why the views of prominent psychiatrists, though widely endorsed by a predominately male medical profession, were subject to greater criticism by female physicians including Mary Putnam Jacobi, whose in depth study 'The Question of Rest in Menstruation' [Jacobi 1876] used quantifiable evidence to point to the contradiction between dominant theories and practice.”  

 2) All humans are hormonal. Somehow only women are thought to be hormonal, and because of that women have often been excluded from biomedical research, because it was believed that their hormonal cycles would influence the results. Also see the excellent work by Rubi et al 2023 in Current Biology. Now the point here is: yes their hormonal status may affect the pharmacokinetics, neurobiology, immunology etc. but that is exactly why we should study it, because otherwise we do not know how women are affected by drugs and illnesses. This is where we currently stand: the awareness that a female body is not just a smaller version of the male body with some additional parts, but that in some aspects it actually works differently.

 By including women we can also improve healthcare for men, because men are not the control condition, men and women need sex-specific health care. We are only at the start of this revolution in medicine. Thank-you for highlighting the benefits to men, we have included this in the introduction as “Yes, accounting for sex-differences is long-overdue and will benefit men as well as women.”. The point that all humans are hormonal we have addressed below.

 3. Ideally in the future we can get rid of the idea that the term hormonal only refers to women.

 1) All animals are hormonal. All sexes have the same sort of hormones, but in different levels.

 2) Besides the sex hormones there are various hormones that play a role in appetite, sleep, energy levels, growth etc.

 3) In all animals hormones fluctuate during the day, as the key role of hormones it to keep us balanced and well equipped to react to changes in the world around us.

 So yes we are all hormonal. Thank you for highlighting this point, which we have incorporated as follows: “The “hormonal female” is a tenacious trope, despite the fact that both male and female bodies produce oestrogen, progesterone and testosterone, plus rely on fluctuation of other hormones such as insulin and thyroid hormone to adaptively regulate their physiology on a daily basis.”
